# Electrocardiographic recording direction impacts ventricular fibrillation waveform measurements: A potential pitfall for VF-waveform guided defibrillation protocols

**DOI:** 10.1016/j.resplu.2021.100114

**Published:** 2021-04-02

**Authors:** Jos Thannhauser, Joris Nas, Priya Vart, Joep L.R.M. Smeets, Menko-Jan de Boer, Niels van Royen, Judith L. Bonnes, Marc A. Brouwer

**Affiliations:** a Department of Cardiology, Radboud University Medical Center, Geert Grooteplein Zuid 10, 6525 GA Nijmegen, The Netherlands; b Department of Health Evidence, Radboud University Medical Center, Geert Grooteplein Zuid 10, 6525 GA Nijmegen, The Netherlands

**Keywords:** Ventricular fibrillation, Amplitude spectrum area, Electrocardiography

## Abstract

**Aim:**

In cardiac arrest, ventricular fibrillation (VF) waveform analysis has identified the amplitude spectrum area (AMSA) as a key predictor of defibrillation success and favorable neurologic survival. New resuscitation protocols are under investigation, where prompt defibrillation is restricted to cases with a high AMSA. Appreciating the variability of in-field pad placement, we aimed to assess the impact of recording direction on AMSA-values, and the inherent defibrillation advice.

**Methods:**

Prospective VF-waveform study on 12-lead surface electrocardiograms (ECGs) obtained during defibrillation testing in ICD-recipients (2010–2017). AMSA-values (mVHz) of simultaneous VF-recordings were calculated and compared between all limb leads, with lead II as reference (proxy for in-field pad position). AMSA-differences between leads I and II were quantified using Bland-Altman analysis. Moreover, we investigated differences between these adjacent leads regarding classification into high (≥15.5), intermediate (6.5–15.5) or low (≤6.5) AMSA-values.

**Results:**

In this cohort (n = 243), AMSA-values in lead II (10.2 ± 4.8) differed significantly from the other limb leads (I: 8.0 ± 3.4; III: 12.9 ± 5.6, both p < 0.001). The AMSA-value in lead I was, on average, 2.24 ± 4.3 lower than in lead II. Of the subjects with high AMSA-values in lead II, only 15% were classified as high if based on assessments of lead I. For intermediate and low AMSA-values, concordances were 66% and 72% respectively.

**Conclusions:**

ECG-recording direction markedly affects the result of VF-waveform analysis, with 20–30% lower AMSA-values in lead I than in lead II. Our data suggest that electrode positioning may significantly impact shock guidance by ‘smart defibrillators’, especially affecting the advice for prompt defibrillation.

## Introduction

Ventricular fibrillation (VF) is the first observed cardiac rhythm in about 20–30% of all out-of-hospital cardiac arrests (OHCAs).^1^, ^2^ In an attempt to improve outcomes, the potential value of VF-waveform analysis is under active investigation.^3^, ^4^, ^5^

At present, the key VF-waveform characteristic is the amplitude spectrum area (AMSA), a combined measure of VF amplitude and frequency, containing predictive value for defibrillation success and survival with favorable neurological outcome.^4^, ^6^, ^7^, ^8^ AMSA correlates with arrest duration and myocardial metabolic state and has been shown to increase during high-quality cardiopulmonary resuscitation (CPR).^9^, ^10^, ^11^ In that context, a current randomised trial compares defibrillation success between the conventional resuscitation protocol and a strategy of defibrillation timing guided by real-time AMSA assessment.^5^ In case of a high AMSA-value (≥15.5 mVHz) the protocol directs immediate delivery of the first shock, while continuation of chest compressions is indicated when AMSA is below 15.5 mVHz.

So-called “smart defibrillators” can automatically perform this real-time analysis of the defibrillator electrocardiogram (ECG). Importantly, previous studies demonstrated that positioning of the defibrillator electrodes may vary greatly in clinical practice.^12^, ^13^ Such variations in bipolar lead recordings could impact AMSA-measurements, which has been suggested in a small surface ECG study.^14^ Depending on the magnitude of this effect, this may impact the abovementioned defibrillation guidance.

In this context, we performed an electrophysiologic study and sought to quantify the impact of recording direction on VF-waveform assessments, in a large series of patients with 12-lead surface ECGs. First, we assessed whether and to what extent AMSA-values vary between different ECG-leads, covering the total spectrum of AMSA values, from low to high. Second, we assessed the impact of ECG recording direction on classification into the currently defined categories of AMSA-values for defibrillation guidance.^4^

## Methods

### Patient population

From our prospective registry of first implantable cardioverter defibrillator (ICD) implantations at the Radboud University Medical Center, we identified all patients with defibrillation testing between 2010−2017. From 2010 to 2013, defibrillation testing was routinely performed in all patients, while in 2014–2017 this was only done for specific indications. For the present analysis, we included all patients with a standard 12-lead surface ECG of induced VF. Exclusion criteria were: age <18 years, congenital heart disease and right-sided ICD implants. Data on demographics, medical history and left ventricular geometry were collected from patient records, as described previously.^15^ Given the observational design of the study, written informed consent was not necessary to obtain according to the Dutch Act on Medical Research involving Human Subjects.

### ICD implantation and testing

**Fig. 1:**
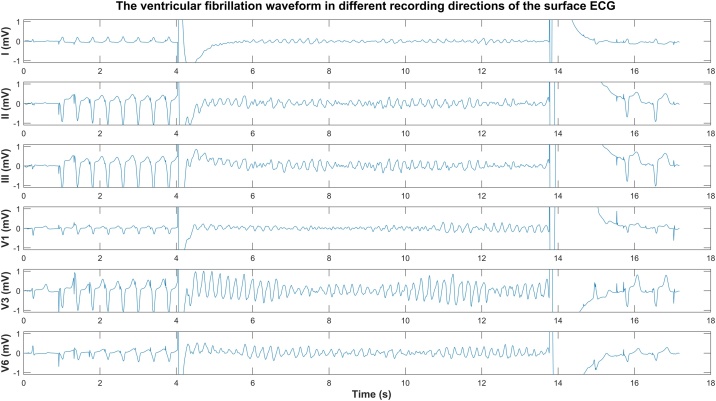
The appearance of the ventricular fibrillation waveform (VF) in six different leads of the surface electrocardiogram. First, VF is induced using a T-wave shock procedure. After about 10 s of VF, the arrhythmia is terminated by an electrical shock of the ICD.

The devices implanted were Medtronic (Minneapolis, Minn, USA), St Jude Medical (St. Paul, Minn, USA) or Biotronik (Berlin, Germany) ICD, cardiac resynchronisation therapy-defibrillator systems with transvenous single coil leads, or subcutaneous ICD-systems (s-ICDs, Boston Scientific, Marlborough, MA, USA). An example of VF induction and termination by an ICD shock is shown in Fig. 1. Defibrillation testing was performed after ICD implantation to test the ability of the implanted device to sense, detect and terminate VF appropriately, according to our local hospital protocol. After sedation with propofol, VF was induced using T-wave shock, direct current pulses or 50 Hz burst pacing (in case of s-ICDs). The presence of VF was confirmed on surface ECG recordings. Transvenous ICDs were programmed to deliver three sequential shocks (15-25-35 Joule) until VF was terminated, while s-ICDs were programmed to deliver two sequential shocks (50–65 Joule, according to the prevailing protocol at the time of study). In case of persisting VF after the third (transvenous devices) or second shock (s-ICDs), external defibrillation was performed.

### VF waveform characteristics

Prior to defibrillation testing, a standard 12-lead surface ECG was attached following standardised protocols, recorded with BARD LabSystem (Lowell, Massachusetts, USA; sampling frequency 1000 Hz; 16-bit A/D converter). For each patient, we studied a single VF-waveform segment prior to the first ICD shock given on VF.

All VF-waveform analyses were performed using Matlab (version 2018a, Mathworks, Natick, MA, USA). VF-signals were pre-processed with a 1−48 Hz bandpass filter. In concordance with the key paper that studied threshold values of AMSA, we analysed VF-segments of 2.05 s (2048 samples)^4^ Subsequently, the signal was converted to the frequency domain using a fast Fourier transform. From the amplitude frequency spectrum, AMSA was calculated as the summed product of individual frequencies and their corresponding amplitudes over an interval of 2−48 Hz, as described previously.^15^, ^16^

### Aims

The primary aim was to quantify the impact of ECG recording directions on the value of AMSA, with particular interest for differences between lead I and II. Analogous to previous studies, lead II was considered as a proxy for the defibrillator ECG recording direction.^16^, ^17^, ^18^, ^19^

Secondly, we aimed to investigate whether and to what extent ECG recording direction affected classification into the currently prevailing AMSA-categories.^4^

### Quantification of impact recording direction

First, we assessed AMSA-values for the bipolar limb leads, augmented leads and precordial leads. Subsequently, we focused on differences observed between lead I and lead II, representing a 60˚ difference in recording direction. Per subject, AMSA-differences (AMSA_diff_ = AMSA_lead I_ – AMSA_lead II_) and AMSA-means (AMSA_mean_ = (AMSA_lead I_ + AMSA_lead II_)/2) were obtained from corresponding, simultaneously recorded VF-segments of leads I and II, and Bland-Altman analysis was performed.^20^ In the resulting scatter plots, AMSA_diff_ is plotted against AMSA_mean_, providing an overview of the magnitude of the observed differences over the total range of AMSA-values.

### Impact on classification into AMSA-categories

All AMSA-values obtained from VF-waveform calculations in lead I and lead II were categorised, based on the three currently prevailing AMSA-categories for defibrillation guidance: high (≥15.5 mVHz), intermediate (6.5–15.5 mVHz) or low (≤6.5 mVHz).^4^, ^5^ We assessed whether classification differed with AMSA-values based on lead I versus lead II.

### Statistics

All statistical analyses were performed with IBM SPSS statistics software (version 25, IBM Corp., Armonk, NY, USA). Categorical variables were presented as numbers (percentages), continuous variables were presented as means ± standard deviations (SD). Comparisons between AMSA-values in different leads were performed using a one way repeated measures analysis of variance (ANOVA). In case of significant differences, post-hoc pairwise comparisons were performed using a paired *t*-test with Bonferroni correction. To assess the level of agreement with regard to classification into AMSA-categories, we used Cohen’s kappa. For all analyses, a p-value of <0.05 was considered statistically significant.

## Results

### Patient characteristics

In total, we studied 243 patients with available ECG-recordings of induced VF, of whom baseline characteristics are reported in Table 1. The mean age was 63 ± 13 years and 77% (186/243) was male. ICDs were implanted for secondary prevention in 38% (92/243), 28% (68/243) were CRT-D devices and 4% (9/243) were subcutaneous ICD-systems. Forty-three percent (105/243) of all patients had a previous myocardial infarction. Twelve percent (30/242) used amiodarone on a daily basis and 89% (215/242) were treated with a beta blocker.

**Table 1:** Baseline characteristics of study cohort with surface ECG-recordings of VF.

Baseline characteristics	
Age (yrs)	63 ± 13
Male gender	186 (77)
Hypertension §	98 (41)
Diabetes §	54 (22)
Atrial fibrillation §	70 (29)
History of myocardial infarction §	105 (43)
Secondary prevention	92 (38)
CRT-D	68 (28)
Subcutaneous ICD	9 (4)
BSA (m^2^) §§	2.0 ± 0.2
LV ejection fraction (%) †	37 ± 14
LV internal diastolic diameter index (cm/m^2^) ††	3.0 ± 0.5
QRS duration (ms) ¶	122 ± 28
Beta blocker §	215 (89)
Amiodarone §	30 (12)

Values are reported as n (%), means ± standard deviations or medians (interquartile ranges), whichever appropriate. BSA: body surface area, CRT-D: cardiac resynchronisation therapy-defibrillator, ECG: electrocardiogram, ICD: implantable cardioverter defibrillator; LV: left ventricular; VF: ventricular fibrillation.

§ Derived from N = 242 patients.

§§ Derived from N = 195 patients.

† Derived from N = 164 patients.

†† Derived from N = 189 patients.

¶ Derived from N = 193 patients.

### VF-waveform characteristics

#### AMSA-values: limb leads

**Fig. 2:**
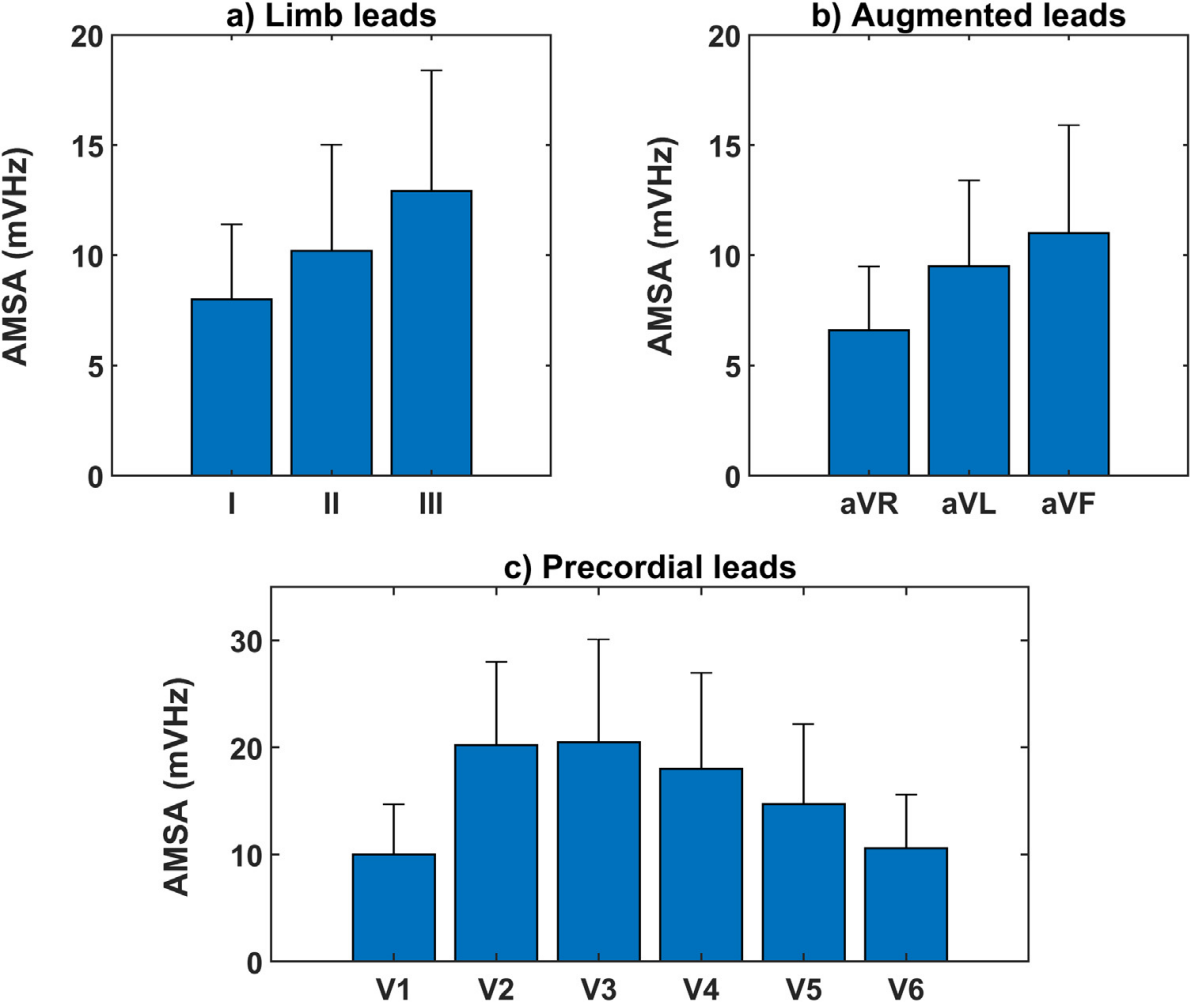
(a) AMSA of limb leads (means + standard deviation). Significant differences were found between AMSA-values (ANOVA p < 0.001). Post-hoc pairwise comparisons revealed significant differences between all lead pairs (all t-tests p < 0.001, p-for-significance after Bonferroni correction = 0.017). (b) AMSA of augmented leads (means + standard deviation). Significant differences were found between AMSA-values (ANOVA p < 0.001). Post-hoc pairwise comparisons revealed significant differences between all lead pairs (all t-tests p < 0.001, p-for-significance after Bonferroni correction = 0.017). (c) AMSA of precordial leads (means + standard deviation). Significant differences were found between AMSA-values (ANOVA p < 0.001). Post-hoc pairwise comparisons revealed significant differences between all lead pairs, except from V1-V6 and V2-V3 (all other t-tests p < 0.001, p-for-significance after Bonferroni correction = 0.008).

Mean AMSA-values obtained from the limb leads of the surface ECG are shown in Fig. 2a. The highest AMSA-values were found in lead III, while the lowest AMSA-values were observed in lead I (12.9 ± 5.5 mVHz vs 8.0 ± 3.4 mVHz, paired *t*-test p < 0.001). The mean AMSA-value in lead II was 10.2 ± 4.8 mVHz, which was significantly different from lead I and lead III (both pairwise tests p < 0.001).

#### AMSA-values: augmented limb leads

Mean AMSA-values obtained from the augmented leads of the surface ECG are shown in Fig. 2b. The highest AMSA-values were found in lead aVF, while the lowest AMSA-values were observed in lead aVR (11.0 ± 4.9 mVHz vs 6.6 ± 2.9 mVHz, paired *t*-test p < 0.001).The mean AMSA-value in lead aVL was 9.5 ± 3.9 mVHz, which was significantly different from lead aVR and lead aVF (both pairwise tests p < 0.001).

#### AMSA-values: precordial leads

Mean AMSA-values obtained from the precordial leads of the surface ECG are shown in Fig. 2c. We observed significant differences between AMSA-values of the precordial leads (ANOVA p < 0.001). The highest AMSA-values were found in lead V3 and the lowest in lead V1 (20.5 ± 9.6 mVHz vs. 10.0 ± 4.7 mVHz, pairwise *t*-test p < 0.001). Significant differences were found between all precordial leads (all p < 0.001), except for lead pairs V1-V6 (p = 0.08) and V2–V3 (p = 0.52).

### Impact of recording direction: Lead I vs. Lead II

**Fig. 3:**
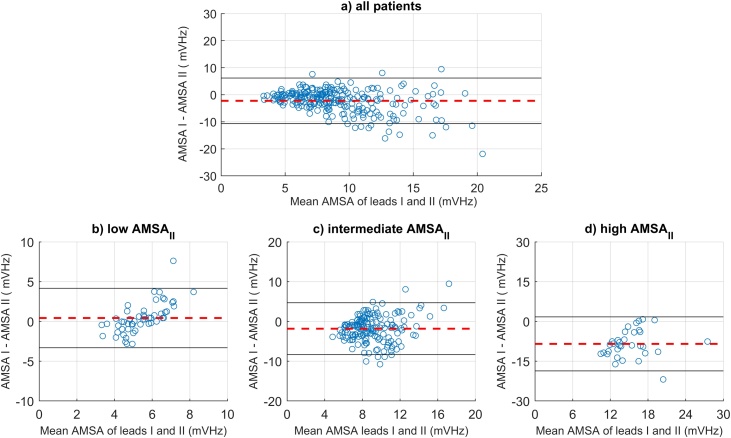
Bland-Altman plots of amplitude spectrum area (AMSA) values, simultaneously acquired by surface ECG lead I and lead II. Figure a: total population; Figure b: patients with a low AMSA in lead II (≤6.5 mVHz); Figure c: patients with an intermediate AMSA in lead II (6.5–15.5 mVHz); Figure d: patients with a high AMSA in lead II (≥15.5 mVHz).

Fig. 3a shows the Bland-Altman plot of AMSA-values of ECG-leads I (AMSA_I_) and II (AMSA_II_). The difference in AMSA between leads I and II (AMSA_I_ – AMSA_II_) was −2.24 ± 4.3 mVHz, corresponding to a relative difference between these leads of −22%. Absolute and relative differences between AMSA-values of lead I and lead II varied per AMSA-category (Fig. 3 b–d). In case of a low AMSA in lead II (≤6.5 mVHz), the mean AMSA was slightly higher in lead I than in lead II (0.4 ± 1.9 mVHz, +8%). For the intermediate and high AMSA_II_ values, the mean AMSA was lower in lead I than in lead II. In case of an intermediate AMSA_II_ (6.5–15.5 mVHz) the difference in AMSA was −1.8 ± 3.3 mVHz (−18%). In case of a high AMSA_II_ (≥15.5 mVHz), the highest absolute and relative differences were found: −8.8 ± 5.2 mVHz (−44%).

### Impact on classification into AMSA-categories

**Fig. 4:**
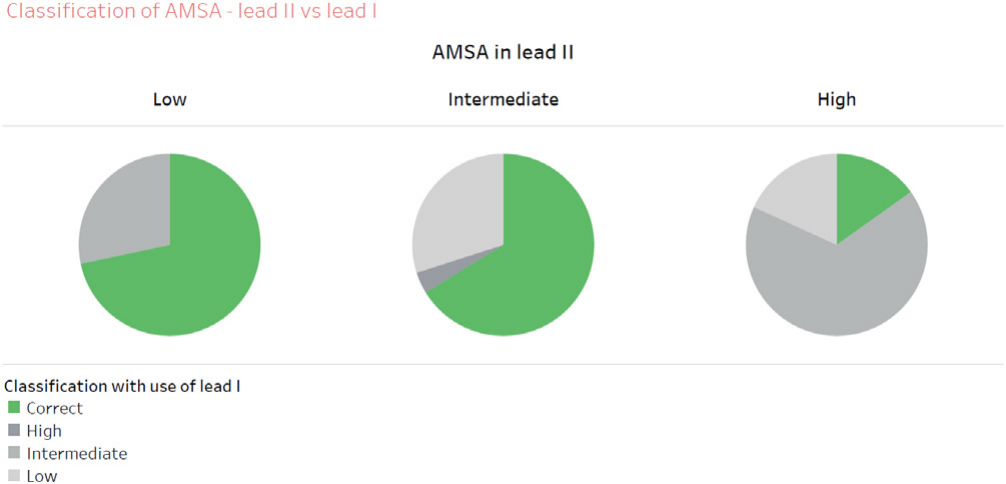
Categorization of AMSA-values when using VF-waveform information of lead I, compared to lead II. AMSA in lead II (mVHz) is categorised as ‘low’ (≤6.5, N = 53), ‘intermediate’ (6.5–15.5, N = 157) or high (≥15.5, N = 33).

Of the 243 AMSA-values obtained from lead II, 53 (22%) were classified into the low, 157 (65%) into the intermediate and 33 (13%) into the high AMSA-category (Fig. 4).

Of the 33 patients with a high AMSA in lead II, 15% had a corresponding high AMSA when calculated from lead I, while 85% was classified as either intermediate (67%) or low AMSA (18%).

Of the 157 patients with an intermediate AMSA in lead II, 66% was also classified as intermediate with data from lead I. For the 53 patients with a low AMSA in lead II, 72% was also classified as low with data from lead I, Cohen’s kappa = 0.27.

## Discussion

In follow-up on VF-waveform studies that reported on shock success prediction^4^, ^21^ and clinical studies demonstrating the prognostic value of VF-waveform analysis,^3^, ^22^ this experimental study was undertaken to provide additional information that may contribute to the further logistic development and optimal application of this promising technology. In this large series, we quantified the impact of ECG recording direction on AMSA over its entire spectrum of values. In relative terms, AMSA differed 20–30% when recorded in a more horizontal direction, with an absolute 2.2 mVHz lower mean AMSA value in lead I than in lead II. Translated to clinical practice during OHCA, calculation of absolute AMSA-values by a smart defibrillator will be subject to electrode placement, resulting in a VF-guided defibrillation advice that will less likely direct towards immediate defibrillation, in case pads are placed more horizontally.

### AMSA and cardiac arrest

In cardiac arrest, AMSA correlates with arrest duration and myocardial metabolic state.^9^, ^18^ This has been regarded the underlying mechanism for AMSA to have moderate to good predictive ability for defibrillation success and favorable neurological outcome.^4^, ^6^, ^7^

Of the potential applications of VF-waveform analysis, shock success prediction has been investigated most extensively.^23^, ^24^, ^25^, ^26^, ^27^, ^28^ In a large OHCA-cohort, threshold values for AMSA to predict shock success have been derived, with classification of AMSA as either *low* (≤6.5 mVHz), *intermediate* (6.5–15.5 mVHz) or *high* (≥15.5 mVHz).^4^, ^5^ Currently, a randomised trial investigates a new resuscitation protocol, based on real-time AMSA analysis by a ‘smart defibrillator’. Shock success with the new resuscitation protocol will be compared to that achieved with a standard CPR-protocol. In the new protocol, prompt defibrillation is only performed when AMSA is high, while the first shock is postponed for additional chest compressions if AMSA is classified as intermediate or low. Moreover, if AMSA reaches a value above 15.5 mVHz during subsequent 2-minute CPR-cycles, the protocol directs immediate defibrillation as well.

In the context of such new resuscitation protocols, applying cut-off values for AMSA, it is a key aspect to study reproducibility of AMSA-measurements. From studies with both laypersons and professionals it is known that pad misplacement is common in clinical practice, resulting in variations in bipolar lead recording.^12^, ^13^, ^29^, ^30^, ^31^ In a previous investigation on the correctness of pad placement in a sample of 136 health care professionals whose duty was to perform defibrillation, only 25% of the subjects attached both pads within 5 cm of the proposed range.^31^ Although misplacement was not further defined in terms of absolute angles, it was shown that pads were predominantly placed either too low (sternal pad) or too medial (apical pad), but variations were present in both vertical as well as horizontal directions. A previous small study (n = 45) already suggested that such variations in recording direction could impact amplitude and frequency related VF-measures.^14^

In this larger patient series we now provide a quantitative estimation of how AMSA-values differ in relation to recording direction, and we studied this aspect over a wide range of values, also including ECG-leads in the transverse plane. On average, we found absolute AMSA-values to be markedly lower in lead I than in lead II (−2.2 ± 4.3 mVHz), with less pronounced differences in lower, and marked differences in higher AMSA values. While AMSA-values were observed to be almost equal in the low category (≤6.5 mVHz, absolute difference 0.4 mVHz, +8%), AMSA in lead I was markedly lower than in lead II in the high category (≥15.5 mVHz, absolute difference −8.8 mVHz, −44%). In relative terms, the observed average difference between lead I and lead II was 22%, which corroborates with the about 30% difference as reported previously.^14^ AMSA-measurements in the transverse plane resulted in clearly higher values than in the coronal plane, which may be related to the fact that leads are located much closer to the myocardial wall.

Translating these experimental findings into potential consequences in the field, we applied our findings to the currently used protocol of so-called ‘smart defibrillators’ during OHCA. In this protocol, prompt delivery of the first shock is advised in case of a high AMSA of ≥15.5 mVHz.^5^

We showed that 85% of the high AMSA-values based on lead II are classified as either ‘intermediate’ or ‘low’ based on lead I. This classification difference shows the marked clinical impact of a more horizontal recording direction, resulting in a lower likelihood that a prompt first defibrillation will be advised. Moreover, in the AMSA-trial, the VF-guided defibrillation threshold of 15.5 mVHz is not restricted to the first, but applies to all shocks, and the placement issue therefore has implications for all subsequent CPR-cycles throughout the resuscitation attempt.

## Implications

The findings of our study provide important information for future directions of new resuscitation protocols with smart defibrillators. The key rationale of such protocols is that a high AMSA is associated with a high chance of shock success. Although the hypothesis to perform early defibrillation in these patients and postpone defibrillations in others may be right, incorrect pad placement may affect AMSA-values. In turn, this may have implications for shock advice, which may ultimately impact trial outcomes. Our current findings may be of value to interpret trial outcomes in a more comprehensive context and provide an explanation why negative studies on such protocols may not necessarily mean that the initial concept can be refuted.

Moreover, from an electrophysiological point of view, this is one of the largest studies on 12-lead VF registrations, providing detailed information on the VF-waveform in various recording directions. This information may contribute to future initiatives with a study design to specifically unravel the quantitative association between recording direction and AMSA assessment, as this may potentially contribute to further refinement of existing algorithms. We observed the highest AMSA-value in lead III of the coronal plane, and in lead V3 of the transverse plane. The fact that AMSA differs across limb leads, suggests that the locality of the VF rotors may contribute to the appearance of the VF-waveform in a certain direction.

Previously, we demonstrated that the presence or absence of prior infarction also affects the VF-waveform, and that AMSA values are lower in the leads adjacent to the area of infarction.^15^ In patients without infarction myocardial mass is another factor that plays a role.^32^

Additional OHCA-studies are warranted to further explore all different aspects involved that potentially affect VF-waveform measurements with smart defibrillators in the OHCA situation.^33^

## Limitations

In terms of inferences to the OHCA-setting, our data reflect early, induced VF. This may limit the generalizability of the observed magnitude of the absolute differences, but the direction of the observed differences will not change. In addition, we studied surface ECG-recordings, and not defibrillator pad registrations. However, from an electrophysiological point of view, the used limb lead registrations are bipolar, just like defibrillator ECG-registrations, which makes it reasonable to assume that the observed differences apply to other devices and in-field VF as well.

Although we calculated AMSA-values in concordance with the key paper on this topic,^4^ technical aspects (e.g. built-in device and filter settings) may have influenced absolute AMSA-values in this study. Yet, for our current study question, addressing within-patient differences between lead I and lead II, this does not affect the main conclusions.

Lastly, we studied the population of ICD-recipients as a whole, and did not stratify our analyses according to underlying cardiac disease. In sub-analyses on the effect of recording direction between patients with and without a previous myocardial infarction, we found uniform results in both subsets.

## Conclusion

In this conceptual VF-waveform study with 12-lead surface ECG-recordings, we demonstrate that AMSA-values are markedly affected by ECG recording direction, with values that are lower in lead I than in lead II, in relative terms 20–30%. Appreciating the variability in pad placement during cardiac arrest, our findings indicate that a more horizontal recording direction leads to lower AMSA-values. For studies on new resuscitation protocols with ‘smart defibrillators’, this implies that the corresponding VF-guided defibrillation will less likely direct towards prompt administration of the first shock. In an era of studies on AMSA-guided defibrillation timing, our findings highlight important aspects for the full interpretation of such trials and may provide new directions for the optimal implementation of 'smart defibrillators' in the setting of out-of-hospital cardiac arrest.

## CRediT authorship contribution statement

**Jos Thannhauser:** Conception and design, Collection and assembly of data, Analysis and interpretation of data, Drafting of the manuscript, Final approval of the manuscript. **Joris Nas:** Conception and design, Analysis and interpretation of data, Drafting of the manuscript, Final approval of the manuscript. **Priya Vart:** Analysis and interpretation of data, Critical revising, Final approval of the manuscript. **Joep L.R.M. Smeets:** Collection and assembly of data, Critical revising, Final approval of the manuscript. **Menko-Jan de Boer:** Collection and assembly of data, Critical revising, Final approval of the manuscript. **Niels van Royen:** Analysis and interpretation of data, Critical revising, Final approval of the manuscript. **Judith L. Bonnes:** Conception and design, Collection and assembly of data, Analysis and interpretation of data, Critical revising, Final approval of the manuscript. **Marc A. Brouwer:** Conception and design, Analysis and interpretation of data, Drafting of the manuscript, Final approval of the manuscript.

The manuscript has not been published and is not being considered for publication elsewhere in whole or in part in any language. All authors have read and approved the manuscript.

## Conflicts of interest

Prof. de Boer is a member of the European advisory board on interventional cardiology of Medtronic. Prof. van Royen received research grants from 10.13039/100001316Abbott, 10.13039/501100005035Biotronik, 10.13039/100004325AstraZeneca and 10.13039/100004320Philips, and professional fees from Abbott and Medtronic. The other authors have no conflicts of interest to declare.

## References

[1] Berdowski J., Berg R.A., Tijssen J.G., Koster R.W. (2010). Global incidences of out-of-hospital cardiac arrest and survival rates: Systematic review of 67 prospective studies. Resuscitation.

[2] Kiguchi T., Okubo M., Nishiyama C. (2020). Out-of-hospital cardiac arrest across the World: First report from the International Liaison Committee on Resuscitation (ILCOR). Resuscitation.

[3] Indik J.H., Conover Z., McGovern M. (2014). Association of amplitude spectral area of the ventricular fibrillation waveform with survival of out-of-hospital ventricular fibrillation cardiac arrest. J Am Coll Cardiol.

[4] Ristagno G., Mauri T., Cesana G. (2015). Amplitude spectrum area to guide defibrillation: a validation on 1617 patients with ventricular fibrillation. Circulation.

[5] Ristagno G., Latini R. Real Time Amplitude Spectrum Area to Guide Defibrillation. ClinicalTrials.gov [Internet]. Identifier: NCT03237910; 2017 Aug 3 [cited 2020 Aug 12] Available from: https://ClinicalTrials.gov/show/NCT03237910.

[6] Indik J.H., Conover Z., McGovern M. (2015). Amplitude-spectral area and chest compression release velocity independently predict hospital discharge and good neurological outcome in ventricular fibrillation out-of-hospital cardiac arrest. Resuscitation.

[7] Schoene P., Coult J., Murphy L. (2014). Course of quantitative ventricular fibrillation waveform measure and outcome following out-of-hospital cardiac arrest. Heart Rhythm.

[8] Coult J., Kwok H., Sherman L., Blackwood J., Kudenchuk P.J., Rea T.D. (2018). Ventricular fibrillation waveform measures combined with prior shock outcome predict defibrillation success during cardiopulmonary resuscitation. J Electrocardiol.

[9] Salcido D.D., Menegazzi J.J., Suffoletto B.P., Logue E.S., Sherman L.D. (2009). Association of intramyocardial high energy phosphate concentrations with quantitative measures of the ventricular fibrillation electrocardiogram waveform. Resuscitation.

[10] Eftestol T., Wik L., Sunde K., Steen P.A. (2004). Effects of cardiopulmonary resuscitation on predictors of ventricular fibrillation defibrillation success during out-of-hospital cardiac arrest. Circulation.

[11] Thannhauser J., Nas J., van Grunsven P.M. (2019). The ventricular fibrillation waveform in relation to shock success in early vs. late phases of out-of-hospital resuscitation. Resuscitation.

[12] Heames R.M., Sado D., Deakin C.D. (2001). Do doctors position defibrillation paddles correctly? Observational study. BMJ.

[13] Bodtker H., Rosendahl D. (2018). Correct AED electrode placement is rarely achieved by laypersons when attaching AED electrodes to a human thorax. Resuscitation.

[14] Indik J.H., Peters C.M., Donnerstein R.L., Ott P., Kern K.B., Berg R.A. (2008). Direction of signal recording affects waveform characteristics of ventricular fibrillation in humans undergoing defibrillation testing during ICD implantation. Resuscitation.

[15] Bonnes J.L., Keuper W., Westra S.W. (2015). Characteristics of ventricular fibrillation in relation to cardiac aetiology and shock success: A waveform analysis study in ICD-patients. Resuscitation.

[16] Thannhauser J., Nas J., Rebergen D.J. (2020). Computerized analysis of the ventricular fibrillation waveform allows identification of myocardial infarction: A proof-of-concept study for smart defibrillator applications in cardiac arrest. J Am Heart Assoc.

[17] Gundersen K., Kvaløy J.T., Kramer-Johansen J., Steen P.A., Eftestøl T. (2009). Development of the probability of return of spontaneous circulation in intervals without chest compressions during out-of-hospital cardiac arrest: an observational study. BMC Med.

[18] Gazmuri R.J., Kaufman C.L., Baetiong A., Radhakrishnan J. (2016). Ventricular fibrillation waveform changes during controlled coronary perfusion using extracorporeal circulation in a Swine model. PLoS One.

[19] Indik J.H., Hilwig R.W., Zuercher M., Kern K.B., Berg M.D., Berg R.A. (2009). Preshock cardiopulmonary resuscitation worsens outcome from circulatory phase ventricular fibrillation with acute coronary artery obstruction in swine. Circ Arrhythm Electrophysiol.

[20] Bland J.M., Altman D.G. (1986). Statistical methods for assessing agreement between two methods of clinical measurement. Lancet.

[21] Nakagawa Y., Amino M., Inokuchi S., Hayashi S., Wakabayashi T., Noda T. (2017). Novel CPR system that predicts return of spontaneous circulation from amplitude spectral area before electric shock in ventricular fibrillation. Resuscitation.

[22] Hulleman M., Salcido D.D., Menegazzi J.J. (2017). Predictive value of amplitude spectrum area of ventricular fibrillation waveform in patients with acute or previous myocardial infarction in out-of-hospital cardiac arrest. Resuscitation.

[23] Povoas H.P., Bisera J. (2000). Electrocardiographic waveform analysis for predicting the success of defibrillation. Crit Care Med.

[24] Marn-Pernat A., Weil M.H., Tang W.C., Pernat A., Bisera J. (2001). Optimizing timing of ventricular defibrillation. Crit Care Med.

[25] Nakagawa Y., Sato Y., Kojima T. (2013). Amplitude spectral area: Predicting the success of electric shock delivered by defibrillators with different waveforms. Tokai J Exp Clin Med.

[26] Coult J., Sherman L., Kwok H., Blackwood J., Kudenchuk P.J., Rea T.D. (2016). Short ECG segments predict defibrillation outcome using quantitative waveform measures. Resuscitation.

[27] Freese J.P., Jorgenson D.B., Liu P.Y. (2013). Waveform analysis-guided treatment versus a standard shock-first protocol for the treatment of out-of-hospital cardiac arrest presenting in ventricular fibrillation: results of an international randomized, controlled trial. Circulation.

[28] Ristagno G., Li Y., Fumagalli F., Finzi A., Quan W. (2013). Amplitude spectrum area to guide resuscitation-a retrospective analysis during out-of-hospital cardiopulmonary resuscitation in 609 patients with ventricular fibrillation cardiac arrest. Resuscitation.

[29] Foster A.G., Deakin C.D. (2019). Accuracy of instructional diagrams for automated external defibrillator pad positioning. Resuscitation.

[30] Nurmi J., Castren M. (2005). Layperson positioning of defibrillation electrodes guided by pictorial instructions. Resuscitation.

[31] Nurmi J., Rosenberg P., Castren M. (2004). Adherence to guidelines when positioning the defibrillation electrodes. Resuscitation.

[32] Bonnes J.L., Thannhauser J., Nas J. (2017). Ventricular fibrillation waveform characteristics of the surface ECG: Impact of the left ventricular diameter and mass. Resuscitation.

[33] Hulleman M., Salcido D.D., Menegazzi J.J. (2020). Ventricular fibrillation waveform characteristics in out-of-hospital cardiac arrest and cardiovascular medication use. Resuscitation.

